# Pneumatic Retinopexy for the Management of Rhegmatogenous Retinal Detachment

**DOI:** 10.7759/cureus.94640

**Published:** 2025-10-15

**Authors:** Konstantinos Zinovios, Sofia Androudi, Athanasios Karamitsos, Emmanouil Mavrikakis

**Affiliations:** 1 First Ophthalmology Department, Ophthalmiatreio Athinon, Athens, GRC; 2 School of Medicine, Aristotle University of Thessaloniki, Thessaloniki, GRC; 3 Ophthalmology, University of Thessaly, Larissa, GRC; 4 Second University Eye Clinic, Aristotle University of Thessaloniki, Thessaloniki, GRC; 5 Ophthalmology, Gennimatas General Hospital, Athens, GRC

**Keywords:** aniseikonia, cataract, metamorphopsia, pars plana vitrectomy, pneumatic retinopexy, rhegmatogenous retinal detachment, visual acuity

## Abstract

Pneumatic retinopexy (PnR) is a minimally invasive, office-based procedure for the management of rhegmatogenous retinal detachment (RRD) in selected patients. Pars plana vitrectomy (PPV) is another common surgical approach for RRD repair, as well as for various other retinal pathologies. PnR is associated with superior functional outcomes, particularly best-corrected visual acuity (BCVA) and metamorphopsia, along with lower rates of cataract progression compared to PPV. This narrative review compares the two techniques, explaining these outcomes. We performed a literature search using PubMed, the Cochrane Library, and Google Scholar to identify articles comparing PnR to PPV. Systematic reviews, meta-analyses, and randomized controlled trials (RCTs), published in the English language within the last 10 years, were included. PnR is associated with lower rates of metamorphopsia than PPV due to reduced retinal displacement and better preservation of photoreceptor integrity. Additionally, factors such as oxygen and light toxicity, aging, lens touch, and tamponade agents contribute to more frequent cataract development following PPV than PnR. Although the primary reattachment rate of PnR is lower than that of PPV, the final reattachment rate is similar. Despite this, BCVA appears to be superior with PnR due to PPV’s stronger association with retinal displacement, outer retinal folds, and discontinuity of outer retinal layers. While PPV is a commonly performed procedure for RRD repair, PnR offers superior functional visual outcomes and a lower risk of cataract progression. Future research, particularly larger RCTs with longer follow-ups, should focus on the quality of vision rather than solely on anatomical success.

## Introduction and background

Rhegmatogenous retinal detachment (RRD) is the most common form of retinal detachment. The annual incidence of RRD is approximately 10 cases per 100,000 individuals, though it may be more frequent in conditions such as older age, myopia, previous ocular surgeries, and trauma [1,2]. In RRD, a retinal break occurs, and liquified vitreous passes through the subretinal space. This leads to the separation of the neurosensory retina from the retinal pigment epithelium (RPE). This sight-threatening condition requires prompt intervention to restore retinal attachment and prevent vision loss. Various techniques are used to repair RRD, including pars plana vitrectomy (PPV), pneumatic retinopexy (PnR), and scleral buckle (SB), either as standalone procedures or in combination [3].

PPV is the most commonly performed procedure by vitreoretinal surgeons worldwide [4]. The technique involves vitreous gel removal through scleral trocars in the pars plana (3 to 4 mm from the limbus, depending on whether the eye is aphakic, pseudophakic, or phakic), using a vitrector. The retina is flattened, usually by draining subretinal fluid (SRF) through the break. Argon laser photocoagulation or cryotherapy is used to seal the retinal breaks and silicone oil (SO) or gas, e.g., sulfur hexafluoride (SF6) or perfluoropropane (C3F8), tamponade the detached retina, allowing the RPE to resorb the remaining subretinal liquid and neurosensory retina to be reattached to the RPE [5]. PPV is considered the dominant surgical approach for the management of RRD and is typically performed in an operating room (OR) by a specialized vitreoretinal surgeon, using specialized equipment.

On the other hand, PnR is a minimally invasive, office-based procedure for the management of RRD. Modern-day PnR was introduced and described in the 1980s as an alternative to SB [6]. It is less time-consuming, requires shorter recovery time, and does not require hospitalization. PnR is also more cost-effective and provides greater lifetime quality-adjusted life years (QALYs) for patients [7]. In this technique, an expandable gas bubble, usually SF6, approximately 0.6 mL, is injected into the vitreous cavity through the sclera after a small amount of aqueous humor (about 0.3 mL) is removed from the anterior chamber (AC). The gas bubble tamponades the retina temporarily, allowing subretinal fluid displacement and absorption [8]. This is achieved by using the physical properties of the gas: buoyancy, which repositions the retina to its physiological position, and surface tension, occluding the retinal break and preventing liquefied vitreous from entering the subretinal space, thus giving the chance to the RPE pump to absorb the SRF [9]. Cryotherapy (in one-step PnR) or laser photocoagulation (in two-step PnR) is used to seal the retinal break. Patients need to maintain a specific head position for approximately one week to allow the gas bubble to tamponade the detached retina [8].

Three techniques of positioning mainly follow PnR. First, “direct-to-break,” where an appropriate head position immediately allows the gas bubble to tamponade the retinal break. This technique relies entirely on the RPE pump to slowly absorb SRF, with the risk of SRF displacement to the fovea. Second, “steamroller maneuver,” which initiates with an immediate face-down position, for four to six hours in macula-on and macula-off cases, respectively. The head is then gradually elevated by 30° every hour until SRF is displaced into the vitreous cavity and the gas bubble seals the break. This effective, but challenging, technique is more forceful and carries the risk of anatomic abnormalities [10]. Lastly, “mini-steamroll” is described as a hybrid approach combining the previous two techniques, starting with 10 minutes of face-down positioning, followed by direct-to-break head positioning. This allows an amount of SRF to move into the vitreous cavity while the remaining fluid is absorbed by the RPE pump [11].

Hillier et al. (2019) described the PIVOT criteria for patient selection in whom PnR can be performed. A single retinal break or a group of breaks in the detached retina must not exceed one clock hour (30°) and must be located above eight and four o’clock of the retina. Additional breaks or lattice degeneration are permissible anywhere in the attached retina. Although PnR has a lower reattachment rate than PPV, it offers better quality of vision and superior visual outcomes, including better best-corrected visual acuity (BCVA), lower rates of metamorphopsia, and aniseikonia. Additionally, PnR is associated with a significantly lower risk of cataract progression and surgery need, compared to PPV [8].

This narrative review aims to compare PnR to PPV, the most commonly used procedure for the management of RRD, and clarify any possible benefits or disadvantages. Specifically, it seeks to explain if and mainly why PnR is possibly associated with better functional outcomes, regarding the postoperative BCVA and metamorphopsia, as well as why it results in a lower rate of cataract progression compared to PPV.

## Review

Methodology

A literature search was performed using PubMed, the Cochrane Library, and Google Scholar, prioritizing systematic reviews and meta-analyses, given that they are a high level of evidence, according to the Evidence-Based Medicine pyramid. Additionally, we included randomized controlled trials (RCTs) due to their objective, unbiased assessment, and prospective design [12].

The search term “Pneumatic Retinopexy” and its corresponding Medical Subject Headings (MeSH) terms were used. We included systematic reviews, meta-analyses, and RCTs that compared PnR to PPV, as well as relevant studies frequently cited within these articles. Articles were restricted to those published in the English language within the last 10 years. Studies focusing on SB or comparing SB with either PnR or PPV were excluded. The search was focused on functional visual outcomes, in particular BCVA and metamorphopsia, as well as cataract progression, rather than merely anatomic success.

Discussion

To our knowledge, the PIVOT trial was the first prospective RCT to compare PnR to PPV, although PnR was described several years earlier. Despite increasing research interest and favorable results from the PIVOT trial and other PnR studies, regarding the lower rate of metamorphopsia, aniseikonia, cataract progression, and the superior BCVA, the adoption of PnR has not significantly increased [13]. Possible reasons include (a) the delayed integration of clinical innovations into routine practice, which typically takes 17-20 years, as surgeons often prefer familiar procedures [14,15]; (b) greater exposure to PPV during fellowship training, as it applies to a wide range of vitreoretinal pathologies, whereas PnR is limited to RRD [16]; (c) improved retinal visualization in PPV due to advancements in technology [16]; (d) the COVID-19 pandemic, which emerged shortly after PIVOT results [13]; (e) the lower primary reattachment rate of PnR compared to PPV, resulting in a greater risk of second procedure necessity [8]; (f) the more challenging recovery due to the requirement of postoperative fixed head position [11]; and (g) a higher professional fee in PPV versus PnR [7].

Metamorphopsia

Metamorphopsia is a visual symptom characterized by distorted image perception, where straight lines appear curved. Patients may experience distortions in the shape, size, or distance of objects. Metamorphopsia can be either vertical or horizontal, affecting the corresponding axis. This abnormal perception may cause unintentional eye movements due to the instability of vision. It can be diagnosed using the Amsler grid, where each eye is tested individually. Patients focus on a central dot and may perceive straight lines as wavy, bent, or missing, as a result of macular pathology [17].

Aniseikonia is a binocular condition in which each eye perceives an image of a different size, frequently leading to dizziness and balance disorders. Aniseikonia can be either optical, resulting from refractive error, or retinal, resulting from macular abnormalities. Retinal aniseikonia can appear as micropsia, where objects appear smaller than their actual size, or macropsia, where objects appear larger. This condition may cause poor depth perception and stereopsis issues [18].

Metamorphopsia is a significant parameter that affects patients’ visual perception and overall satisfaction. While BCVA remains the primary metric for visual outcomes, visual distortions such as metamorphopsia and aniseikonia can significantly impact daily activities, even in cases with good visual acuity (VA). Several studies suggest that the incidence of metamorphopsia differs between PnR and PPV, potentially due to factors such as retinal displacement and photoreceptor integrity. The goal of RRD repair is to prevent retinal displacement; when displacement is undetectable, it is referred to as high-integrity retinal attachment (HIRA), where the final position of photoreceptors is close to their original position relative to RPE. In contrast, notable retinal displacement is referred to as low-integrity retinal attachment (LIRA), where retinal vessel printings (RVPs) are present [19].

The PIVOT trial demonstrated that PnR was associated with less vertical metamorphopsia at the 12-month follow-up compared to PPV [8]. Additionally, increased aniseikonia was observed in patients treated with PPV [20]. PnR, while associated with a lower primary success rate, appears to preserve visual function more effectively than PPV. PnR is more respectful to the tissues and less invasive than PPV, leading to reduced retinal displacement and better preservation of photoreceptor integrity [21]. It is indicated that RVP, as a sign of retinal displacement, is less severe and occurs less frequently in PnR patients (7%) than in patients who underwent PPV (44.4%) [22]. However, PPV may still offer advantages in macula-on detachments, where the fovea remains unaffected by the detachment itself, though PnR outcomes are not affected by foveal status [21,23].

The reattachment process may result in anatomical and functional abnormalities of the photoreceptors, particularly due to the effects of intraocular tamponade. Several hypotheses have been proposed to explain the possible less metamorphopsia incidence in PnR compared to PPV.

One key factor is retinal displacement following the procedure. Postoperative metamorphopsia is closely linked to the presence and extent of retinal displacement. The percentage of eyes with retinal displacement is lower in PnR than in PPV, and this group achieves better results regarding visual distortions [20]. This difference after successful RRD repair is likely due to the more natural reabsorption of SRF by the RPE pump, the smaller buoyant force, and reduced retinal stretch in PnR, which involves a much smaller gas bubble compared to PPV [21]. Postoperative positioning and tamponade agents also play crucial roles in this process. SRF flow can stretch the thin and elastic retina, usually leading to downward reattachment, LIRA, and vertical metamorphopsia, proportional to the severity of the stretch. SRF is displaced away from the tamponade agent, accumulating in subretinal spaces depending on the gravity and the head position [19]. An immediate face-down position is recommended because SRF moves in all directions rather than settling inferiorly, helping to prevent retinal displacement. In PPV, a fully gas-filled vitreous cavity exerts a stronger buoyant force on the retina compared to SO in PPV or the smaller gas bubble used in PnR [24].

Another hypothesis examined is the discontinuity of the ellipsoid zone (EZ) and external limiting membrane (ELM). Outer retinal microstructural anatomy is significant, as the abnormalities in orientation of photoreceptors with the RPE are associated with metamorphopsia, following successful RRD repair [25]. Central 3-mm and 6-mm foveal scans have shown higher rates of EZ and ELM discontinuity in PPV than in PnR. The forceful evacuation of the subretinal space from the SRF in PPV (through the retinal break or retinotomy), air-fluid exchange, fluid turbulence, and maximal vitreous cavity fill with gas or SO make PPV a more aggressive and active method. In contrast, the passive nature of PnR allows for a more physiological reattachment, reducing stress on retinal cells and minimizing the likelihood of outer retinal microstructural abnormalities and photoreceptor apoptosis [26].

Cataract

Cataract formation is a well-documented and one of the most common postoperative complications following RRD repair. The incidence of cataract progression and the need for surgery are significantly higher in PPV compared to PnR [21]. The PIVOT trial demonstrated that 65% of phakic patients treated with PPV had their cataract extracted before the 12-month follow-up, compared to 16% of phakic patients who underwent PnR [8]. While PnR is an office-based procedure that preserves the vitreous, PPV is an invasive surgery that involves vitreous removal, accelerating lens opacification through multiple mechanisms. This almost inevitable complication in patients undergoing PPV can impact VA, reduce overall satisfaction, and often necessitate a second surgery for cataract extraction. Cataract surgery in vitrectomized eyes or in cases where PPV and phacoemulsification are combined presents additional challenges. The absence of the vitreous reduces posterior segment support, leading to intraoperative instability. Without this counterpressure, unintentional deepening of the AC can occur, increasing the risk of complications during phacoemulsification [27,28].

Multiple mechanisms contribute to cataract formation after PPV, many of which are either absent or have minimal impact in the PnR procedure. Oxygen toxicity, aging, light toxicity, accidental intraoperative lens touch, and tamponade agents have been implicated in cataract pathogenesis. Furthermore, while factors such as the type of irrigation solution and the duration of surgery have been considered, they appear to have no significant impact [27].

The crystalline lens is normally protected from excessive oxygen exposure, as the vitreous maintains the oxygen levels higher near the retina and lower anteriorly. This hypoxic environment protects the lens from oxygen toxicity. However, during PPV, when the vitreous is removed and replaced with balanced salt solution (BSS), oxygen is evenly distributed throughout the vitreous cavity, exposing the lens to oxidative stress. This leads to protein oxidation and nuclear sclerosis [27,29].

Age is another significant factor in cataract formation after PPV. Approximately 80% of patients over the age of 50 years develop cataract postoperatively, whereas younger patients have significantly lower rates of cataract formation [28,30]. The need for cataract extraction surgery doubles with every five-year increase in the patient’s age at the time of undergoing PPV [31].

Another proposed mechanism involves light toxicity to the lens from the operating microscope or the fiber optic probe used during PPV. The lens is normally protected from photo-oxidative damage by the cornea, antioxidants provided by the aqueous humor, and the hypoxic environment of the vitreous. However, photo-oxidative stress and the increased intraocular temperature during surgery may damage lens proteins and lipids, accelerating cataract development [27]. As discussed, PnR is an office-based method for RRD repair, performed without a microscope or intraocular light, eliminating the risk of light toxicity [8].

Lens contact during PPV, which is performed in an OR, is a recognized intraoperative complication that can lead to rapid postoperative cataract formation. Accidental lens touch has been associated with nuclear sclerosis, posterior subcapsular, white, or mixed cataract, often requiring earlier phacoemulsification compared to cases without lens touch. Complicating surgeries, including those involving proliferative retinopathy (PVR), retinectomy, or the use of SO as a tamponade agent, increase the risk of lens touch due to the need for more frequent and closer to the lens manipulations in the vitreous cavity [32].

Tamponade agents, including intraocular gases (SF6, C3F8) and SO, also contribute to cataract development after PPV surgery. Contact between intraocular gases and the lens can lead to posterior capsular and cortical opacities, with the severity depending on the duration of contact and the gas bubble size. The gas bubble is smaller in PnR compared to the fully gas-filled vitreous cavity in PPV, potentially reducing the risk of these opacities [22]. While early-stage opacities may be reversible, prolonged exposure makes them irreversible and frequently results in gradual nuclear sclerosis [30,33,34]. Similarly, SO is associated with cataract formation, particularly when retained in the vitreous cavity for extended periods [35]. Additionally, it is believed that SO induces metabolic changes in the lens capsule, promotes cellular entry, and triggers apoptotic processes through apoptotic factors, leading to lens opacification [27,36].

Visual acuity

In comparing the visual outcomes of PnR and PPV for the treatment of RRD, many studies have evaluated BCVA improvements. While both procedures effectively restore retinal attachment, differences in functional visual outcomes have been reported.

Although PnR is associated with a lower primary reattachment rate (approximately 80%), the final success rate is equals after secondary PPV in failed cases, compared to PPV (90% success rate) [21,37]. Pecaku et al. (2024) concluded in a post hoc analysis that the primary reattachment rate was approximately the same at 12 months, and the BCVA was superior in the PnR group versus the PPV group [23]. In addition, long-term redetachment rates between the two procedures appear to show no statistically significant difference two years after RRD repair [38]. However, many RCTs suggest that VA may be superior with PnR. Hillier et al. (2019) in an RCT involving 172 patients, demonstrated that the postoperative Early Treatment Diabetic Retinopathy Study (ETDRS) letter score, regarding the VA, was better in the PnR group at 3, 6, and 12 months compared to PPV [8]. Additionally, the 25-Item National Eye Institute Visual Function Questionnaire (NEI VFQ-25) [39] was also used to assess vision-related quality of life (VRQoL) and revealed higher scores favoring PnR in the first three and six months postoperatively, but comparable results between the two groups at 12 months [8]. Similarly, Muni et al. (2020) in a trial with 160 patients found that those treated with PnR had superior vision-related functioning scores, compared to the PPV group. In this trial, NEI VFQ-25 had also superior results in six months [40].

Several propositions have been made and examined to explain the better VA in PnR compared to PPV. One of these is retinal displacement, which occurs more frequently in PPV than in PnR. After PnR, patients tend to achieve better BCVA (Snellen 20/30) compared to those undergoing PPV (Snellen 20/44) [23]. However, Mason et al. (2022), in their systematic review and meta-analysis, found no clear relationship between retinal displacement and BCVA [24].

Another possible explanation for superior VA in PnR is the incidence of postoperative outer retinal folds (ORFs). Patients with ORFs had worse ETDRS VA letter scores, with a mean loss of 9.4 letters, compared to those without ORFs at one-year follow-up. PnR was associated with a lower risk of ORF development, possibly due to the more natural reattachment following the slower SRF reabsorption, compared to PPV. On the contrary, the more forceful reattachment in PPV can lead to structural damage, photoreceptor misalignment, and apoptotic photoreceptor cell death [41].

Lastly, the discontinuity of outer retinal layers, particularly the EZ and ELM, can significantly impact VA following RRD repair. Disruptions in these retinal layers have been associated with lower BCVA after successful reattachment. Studies have shown that patients with continuous EZ and ELM structures have a Snellen score of 20/25, whereas those with discontinuous EZ and ELM present with a lower Snellen score of 20/40. Patients undergoing PnR are favored with a less frequent incidence of these abnormalities, possibly due to the slower, more natural reattachment of the retina, which causes less stress to the cells compared to PPV. This gentler approach in PnR may contribute to preserving the structural integrity of the EZ and ELM, minimizing the risk of negative visual outcomes associated with photoreceptor misalignment [26].

Nevertheless, some publications suggest otherwise, indicating that the difference in VA is not statistically significant. A meta-analysis by Zajner et al. (2024), which included three trials, concluded that there was no statistically significant difference in VRQoL between PnR and PPV [42]. Likewise, Karam et al. (2023), analyzing four retrospective case series and one RCT, also reported no significant difference in the improvement of VA between patients who underwent PnR and those who underwent PPV [43].

Strengths and limitations

The strengths of this review include its citation of high-quality evidence, such as systematic reviews, meta-analyses, and RCTs. To our knowledge, this is the first literature review to compare key functional outcomes (metamorphopsia, BCVA) and a common complication of RRD repair (cataract development) between PnR and PPV.

However, several limitations of this review should be noted. Some of the included data come from retrospective and non-randomized trials, which may reduce the strength of conclusions. As a narrative review, it lacks the strict methodology of systematic reviews. The available data comparing PnR to PPV are limited, including details on severity and preoperative BCVA, partly because PnR is not widely performed outside of a few countries, primarily Canada. Additionally, comparisons with SB were excluded, even though SB may be a superior treatment option in specific cases. Finally, the review is restricted to articles published in the English language, which may limit the extent of the findings.

## Conclusions

RRD is a sight-threatening condition that concerns a lot of ophthalmologists, patients, and researchers. PnR and PPV are two techniques for RRD repair. They differ in surgical approach, visual outcomes, and complications. While PnR is associated with a lower primary success rate, its final success rate is comparable to that of PPV. Although retinal reattachment can be achieved with both procedures, the quality of vision remains uncertain. Surgeons should focus more on functional outcomes and not exclusively on anatomical success. PPV is a frequently performed technique in clinical practice, while PnR offers some advantages, including reduced metamorphopsia, lower cataract development rates, and better BCVA in selected patients. Therefore, PnR should be considered a first-line option for these patients. However, the available data are limited, and additional RCTs with longer follow-up periods are needed to further evaluate its benefits. Future research should also focus on detailed visual outcomes, including contrast sensitivity, long-term complications, and postoperative distortions, to provide a more comprehensive comparison between these procedures.
